# Qualitative research to inform economic modelling: a case study in older people’s views on implementing the NICE falls prevention guideline

**DOI:** 10.1186/s12913-021-07056-1

**Published:** 2021-09-28

**Authors:** Joseph Kwon, Yujin Lee, Tracey Young, Hazel Squires, Janet Harris

**Affiliations:** 1 School of Health and Related Research, University of Sheffield, Regent Court (ScHARR), 30 Regent Street, Sheffield, England S1 4DA; 2 Warwick Medical School, University of Warwick, Gibbet Hill Road, Coventry, England CV4 7AL

**Keywords:** Falls, Falls risk, Falls prevention, National Institute for health and care excellence guideline, Implementation, Qualitative research, Facilitators and barriers, Economic model, Public health

## Abstract

**Background:**

High prevalence of falls among older persons makes falls prevention a public health priority. Yet community-based falls prevention face complexity in implementation and any commissioning strategy should be subject to economic evaluation to ensure cost-effective use of healthcare resources. The study aims to capture the views of older people on implementing the National Institute for Health and Care Excellence (NICE) guideline on community-based falls prevention and explore how the qualitative data can be used to inform commissioning strategies and conceptual modelling of falls prevention economic evaluation in the local area of Sheffield.

**Methods:**

Focus group and interview participants (*n* = 27) were recruited from Sheffield, England, and comprised falls prevention service users and eligible non-users of varying falls risks. Topics concerned key components of the NICE-recommended falls prevention pathway, including falls risk screening, multifactorial risk assessment and treatment uptake and adherence. Views on other topics concerning falls prevention were also invited. Framework analysis was applied for data analysis, involving data familiarisation, identifying themes, indexing, charting and mapping and interpretation. The qualitative data were mapped to three frameworks: (1) facilitators and barriers to implementing the NICE-recommended pathway and contextual factors; (2) intervention-related causal mechanisms for formulating commissioning strategies spanning context, priority setting, need, supply and demand; and (3) methodological and evaluative challenges for public health economic modelling.

**Results:**

Two cross-component factors were identified: health motives of older persons; and professional competence. Participants highlighted the need for intersectoral approaches and prioritising the vulnerable groups. The local commissioning strategy should consider the socioeconomic, linguistic, geographical, legal and cultural contexts, priority setting challenges, supply-side mechanisms spanning provider, organisation, funding and policy (including intersectoral) and health and non-health demand motives. Methodological and evaluative challenges identified included: incorporating non-health outcomes and societal intervention costs; considering dynamic complexity; considering social determinants of health; and conducting equity analyses.

**Conclusions:**

Holistic qualitative research can inform how commissioned falls prevention pathways can be feasible and effective. Qualitative data can inform commissioning strategies and conceptual modelling for economic evaluations of falls prevention and other geriatric interventions. This would improve the structural validity of quantitative models used to inform geriatric public health policies.

**Supplementary Information:**

The online version contains supplementary material available at 10.1186/s12913-021-07056-1.

## Background

Falls among older people impose significant morbidity and mortality burdens [1]. Around 30% of community-dwelling persons aged 65+ fall each year [2]. Falls can result in fatal or debilitating injuries such as hip fractures [3], provoke fear of further falls [4], and induce functional decline [5]. They also impose substantial burdens on care systems through hospitalisations and long-term care admissions [6] and on informal caregivers [7]. Falls prevention is hence a public health priority [8].

The rationale for intervention is further supported by randomised controlled trial (RCT) findings that diverse community-based falls prevention interventions significantly reduce the number of falls and fallers [9, 10]. In England and Wales, the National Institute for Health and Care Excellence (NICE) clinical guideline 161 (CG161) is the normative reference point for local clinical practice [2]. This recommends that persons aged 65+ receive falls risk screening at routine visits to health and social care professionals; those screened to be at high risk would then be referred to multidisciplinary falls risk assessment and tailored treatments, including exercise, home assessment and modification (HAM), medication modification and vision improvements [2]. These treatments may also be delivered individually as single-component interventions [11–13], either as substitutes for the multifactorial intervention or as non-mutually exclusive complements [14, 15]. These interactions between screening and treatment components, the multifactorial risk profile of falls as a geriatric syndrome [16], and the wider environmental risk factors [17, 18] introduce substantial complexity to falls prevention [19, 20].

Due to this complexity, community-based falls prevention strategies face significant implementation challenges [21–24]. For example, a recent survey of English GPs found that only 31% routinely screened their older patients for falls history; the median annual number of referrals to falls prevention services per GP was just 10 [25]. Implementation quality can be suboptimal even in RCT settings. For example, the uptake rate for a UK trial of falls prevention exercise was 6% [26]; adherence to different components of multifactorial interventions is as low as 28% [27]; and 16% of participants withdraw from falls prevention exercise at trial conclusion [28]. Low implementation reduces the effectiveness and population reach/impact of falls prevention [20]. Accordingly, NICE CG161 incorporated a systematic synthesis of older people’s views on the facilitators and barriers to falls prevention (covering the period 1990–2003), but found no study that explored their views on multifactorial packages (p. 101) [2]. More recent qualitative works have likewise focused on specific components of the falls prevention pathway, including receptiveness to falls prevention advice [29], falls risk assessment [30], and exercise uptake [31, 32] and adherence [33]. This is an important evidence gap given that complexity results from the interaction of facilitators and barriers across different pathway components. A more holistic approach to qualitative research with current or potential falls prevention service users is warranted.

Health economic evaluation is a comparative analysis of alternative healthcare strategies in terms of costs and consequences with the purpose of informing the efficient use of scarce resources under a constrained healthcare budget [34]; it can also incorporate further decisional criteria beyond cost-effectiveness, such as reduction in social inequities of health, according to stakeholder preference [35–37]. One vehicle for economic evaluation is decision modelling that represents the key causal mechanisms of a decision problem in mathematical and statistical/probabilistic relationships [34]. Decision models are particularly well-suited for considering all relevant costs and effects of interventions over long time horizons, and for evaluating ‘what-if’ scenarios for the full target population of the decision-making jurisdiction [38]. One such scenario is the commissioning of implementation resources to change current local practice into a form approaching the NICE-recommended pathway.

A de novo economic model is likely required if the existing economic models or evidence are insufficient for informing local decision-making: e.g., due to unrealistic representation of local practice and/or shortcomings in characterising the key causal mechanisms. Currently, the decision model developed to inform CG161 [39] evaluates a multifactorial intervention for the national population and may not be locally generalisable; while the locally applicable Public Health England Return on Investment tool [11] only evaluates single-component interventions. This presents a rationale for developing a de novo model evaluating the cost-effectiveness relative to current practice (and wider decisional outcomes) of a strategy that locally implements the NICE-recommended pathway.

Qualitative research with current and potential consumers of health services can contribute to economic modelling in two important ways [40, 41]: (a) eliciting appropriate commissioning strategies; and (b) understanding the key methodological and evaluative challenges to *public health* economic modelling.

Concerning (a), the model-evaluated commissioning strategy should fully reflect the complex network of intervention-related casual mechanisms influencing implementation. Several frameworks exist to capture such complexity [40], including the Context and Implementation of Complex Interventions (CICI) framework [20] which was developed as part of the INTEGRATE-HTA project to consider a comprehensive set of factors influencing the assessment of complex health technologies [19]. CICI distinguishes between contextual factors (e.g., socio-cultural, legal) and implementation mechanisms (e.g., professionals, organisations) that shape implementation quality. Priority-setting challenges – e.g., reducing social inequities of health [35] – also arise from the implementation context [40]. Given the CICI framework’s lack of focus on demand-side mechanisms (e.g., motivations of the older persons to engage in healthy behaviour [42]), it could be supplemented by the health needs assessment (HNA) framework that incorporates demand, supply and need/eligibility as distinct yet overlapping domains [43]. Inductive qualitative data analysis could commence with themes sourced from this combined framework, and thereafter interact with new themes emerging from the data to arrive at the final thematic framework informing the commissioning strategies [44, 45].

Concerning (b), the nature of falls being a *public health* problem faced by a broad spectrum of older populations – rather than a clinical problem faced by a well-defined, narrow patient group – presents further complexity to model development [41]. According to a systematic methodological review, the key methodological challenges to public health economic modelling include: (i) capturing non-health outcomes and societal intervention costs; (ii) considering dynamic complexity in health determinants and intervention need; (iii) considering theories and models of human behaviour based on psychology and sociology; and (iv) considering social determinants of health and issues of equity [46]. Addressing such challenges is part of the INTEGRATE-HTA recommendations (see chapter 3) [19], and is necessary for improving the structural validity of the decision model [41]. The same inductive analysis can identify how these challenges relate to the local decision problem and hence to the decision model structure [41].

In all, a de novo qualitative study of older people is warranted, first to holistically explore the facilitators and barriers for implementing the NICE-recommended falls prevention pathway, and second to proactively use the resulting qualitative data to inform economic modelling. The latter would improve upon the siloed approach that is widely prevalent in the literature, whereby qualitative research is conducted and interpreted separately from economic evaluation, even when both designs are included in the same project [39, 47, 48].

## Aim and objectives

The study aims to capture the subjective views of older people on implementing the NICE CG161 guideline on community-based falls prevention and use the qualitative data to inform the development of a conceptual falls prevention economic model. The latter would guide commissioning decisions in a local health economy seeking to implement CG161, Sheffield being one such setting. The research objectives are to:

1. Identify the facilitators and barriers for implementing key components of the CG161 community-based falls prevention pathway – including falls risk screening and assessment, falls risk awareness, and uptake and adherence of treatments within multifactorial intervention – and contextual factors influencing the pathway implementation in Sheffield.
2. Inform potential local commissioning strategies on falls prevention by understanding the causal mechanisms in context, supply, need and demand that influence implementation.
3. Identify the methodological and evaluative challenges associated with developing a public health economic model of falls prevention in the local context.

Given the aim of informing a model applicable to a local health economy, the identified qualitative themes would likely be locally specific. Hence, the main target audience (outside of Sheffield) are economic modellers and qualitative researchers (and commissioners sponsoring them) interested in applying the methodology used in this case study to other local health economies and public health areas. That said, the facilitators and barriers identified under the first objective would be generalisable to other urban community settings in England and Wales and hence be of interest to professionals and patient groups seeking to improve the implementation of local falls prevention.

## Methods

The qualitative research involved focus groups and interviews with older persons living in the community. The ethics approval was obtained from the Research Ethics Committee at the School of Health and Related Research, University of Sheffield (ref. 025248). Written consent was obtained from willing participants.

### Target population and sampling

**Fig. 1:**
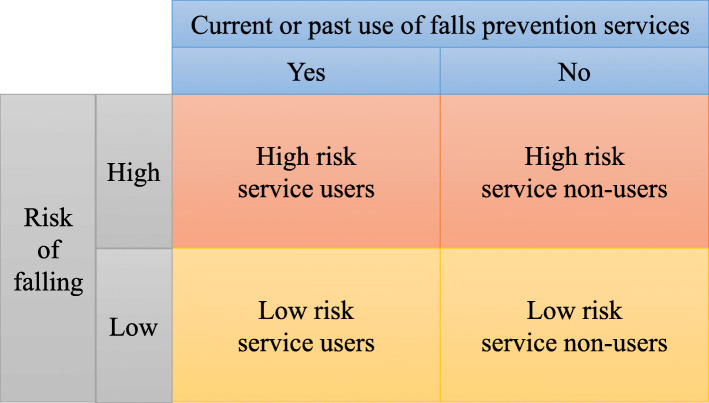
Categories for study participant characteristics

The target population comprised persons aged 65+ in Sheffield, England, and persons aged 50–64 who are at high falls risk. The latter group was included to explore the rationale for earlier prevention as is currently recommended for inpatient settings by CG161 [2]. Purposive sampling covered multiple categories of participant characteristics in terms of falls risk and service use as illustrated in Fig. 1.

According to CG161, those with a history of fall(s) requiring medical attention or recurrent falls in the past year and/or mobility and balance problems were defined as high-risk [2]. Low-risk individuals were sampled because they are still eligible for falls risk screening and/or interested in early prevention.

Recruitment continued until all participant categories were covered and themes saturated. Specifically, two focus groups (FG1, FG2) were formed from two separate cohorts enrolled in Dance to Health, a falls prevention programme that combines evidence-based Otago and Falls Management Exercise components in dance routines [49, 50]; these groups contained high and low risk service users. Two further groups (FG3, FG4) were formed from a Patient and Public Involvement group meeting regularly at the Northern General Hospital and a social group meeting at Zest Community, a local social enterprise offering leisure, health and work support services to diverse age groups; these contained high and low risk service non-users. Two interview participants were recruited from Dance to Health and Zest Community.

Focus groups were held directly before/after the regular meetings. Community organisation staff confirmed before research commencement whether their members could give informed consent. One participant declared memory problems while another a recent diagnosis of Alzheimer’s disease; but both were regular attendees of community groups and expressed confidence in participating. After obtaining written consents, questionnaires were administered to collect data on demographics, falls history and fear of falling, current physical activity, and contact with falls prevention services.

Focus group participants were previously acquainted from attending the same activity and were comfortable sharing their experiences in the group. The main interviewer (JK) introduced himself and his PhD project aim and presented himself as someone wanting to learn from the participants. Participants were motivated to help the interviewer understand their perspective on falls and falls prevention. For interviews, around 15 min were spent for the participants and the interviewer to become acquainted in conversing (at interviewees’ homes) before the research commenced.

### Discussion topics

The main discussion topics were structured around the sequential steps of the proactive prevention pathway recommended by CG161 [2], namely: (i) falls risk screening/assessment by professionals; (ii) participant suggestions on raising falls risk awareness in the community; (iii) initial uptake of different treatments; and (iv) long-term adherence to treatments. The pathway is proactive in that it is initiated by professional referral of high-risk individuals after falls risk screening. If mentioned by participants, two further pathways were discussed: the reactive pathway – where older persons are referred to falls prevention by professionals after medical attention for a fall, which is also recommended by CG161 (see recommendations 1.1.2.1, 1.1.3.2 and 1.1.6.1) [2]; and the self-referred pathway – where older persons enrol in falls prevention without professional referral.

**Fig. 2:**
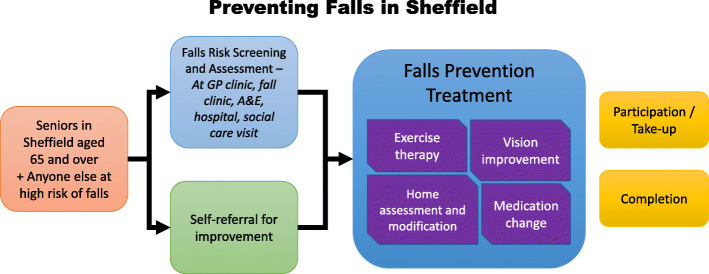
Graphical summary of the recommended falls prevention guideline used to introduce the discussion topics to focus group and interview participants

A simplified graphical summary of the proactive pathway, as shown in Fig. 2, was used to explain the main topics to participants. Four treatment types – exercise, HAM, medication change and vision improvement – were explained while emphasising that other types exist, such as chiropody. It was also highlighted that a reactive pathway after a serious fall is commonly used, and that a self-referred pathway is recommended by experts [51]. Further contextual factors influencing falls risk and prevention (e.g., safety of pedestrian walks in Winter) were actively explored as they emerged during discussion.

### Data collection

Recorded audio data were transcribed and anonymised. The questionnaire data were similarly transferred to an Excel spreadsheet and anonymised. Both data were stored securely in the University designated folder.

### Data analysis

A framework analysis was employed for the analysis of obtained data [44, 45]. The approach involved five stages: (a) familiarisation – which involves repeated listening to audio and reading of transcripts for immersion in the data; (b) identifying a thematic framework – which is based on an a priori set of issues related to the research objectives and themes emerging from the data; (c) indexing – which systematically applies the thematic framework to the transcripts; (d) charting – which ‘lifts’ the data from the transcripts and rearranges them (e.g., in a tabular format) according to the thematic framework; and (e) mapping and interpretation – which seeks associations and develops policy-related strategies from the charted data based on a priori issues and emerging themes. Stages (a) to (c) were conducted independently by two authors (JK and YL). All authors contributed to stages (d) and (e).

From stage (b) onwards, three frameworks related to the research objectives were constructed using a priori concepts and themes emerging from the data:

(I) Framework to understand the facilitators and barriers to components of the NICE CG161 falls prevention pathway and cross-component and contextual factors.
(II) Framework to inform potential commissioning strategies by accounting for causal mechanisms in context, priority setting, need/eligibility, supply and demand.
(III) Framework to understand the key methodological challenges to public health economic model development.

#### Framework (I): facilitators and barriers and cross-component and contextual factors

This framework closely followed the structure of the discussion topics and charted the main themes identified from the data. Facilitators and barriers for the pathway implementation that emerged from the data were arranged by a priori thematic categories corresponding to the NICE CG161 pathway components – i.e., (i) falls risk screening/assessment by professionals; (ii) raising falls risk awareness; (iii) initial uptake of treatments; and (iv) long-term adherence to treatments. Cross-component factors – i.e., facilitators and barriers influencing multiple pathway components – were highlighted. Additional contextual factors influencing the pathway implementation were noted as they emerged from the data.

#### Framework (II): potential commissioning strategies

This framework rearranged the main themes under Framework (I) into a format that guides commissioning strategies (actual or model-evaluated). An a priori CICI-HNA framework was constructed that combined the thematic categories within the CICI [20] and the HNA frameworks [43]. This is illustrated in Fig. A in Supplementary Material with accompanying descriptions. In brief, the CICI framework distinguished between implementation context (e.g., socioeconomic, legal) and mechanisms (e.g., provider, funding) [20]. The HNA framework distinguished between supply, demand and need/eligibility [43]: supply corresponded to the CICI implementation mechanisms; demand encompassed personal and external factors influencing uptake/adherence decisions (e.g., health-related motives for healthy behaviour [42], community marketing, self-efficacy promotions [52, 53]); need/eligibility was determined by normative clinical and public health guidelines and intervention studies that demonstrated a group’s ability to benefit from an intervention [43]. Further thematic categories that emerged from the data were noted (e.g., priority setting challenges that contextualised commissioning [35]). The mapped themes informed commissioning strategies by highlighting which CICI-HNA factors were modifiable – i.e., lie within the decision space which is defined by the stakeholders involved, decision time horizon and budget/capacity constraints – and to what extent.

#### Framework (III): challenges for public health economic modelling

The thematic categories of key methodological challenges for public health economic modelling were taken from a systematic methodological review [46]: (i) capturing non-health outcomes and societal intervention costs; (ii) considering dynamic complexity in health determinants and intervention need; (iii) considering theories and models of human behaviour based on psychology and sociology; and (iv) considering social determinants of health and issues of equity. Additional challenges associated with economic modelling and evaluation were also identified from the emerging data.

## Results

### Participant characteristics

Twenty-seven persons participated in research across four focus groups (FG1–4) and two interviews (INT1–2) between October 2019 and January 2020. Table 1 summarises their characteristics.

**Table 1:** Summary of participant characteristics

Field	Variable		N (%)
Demographics	Sex	Female	20 (74)
		Male	7 (26)
	Age	< 60	5 (19)
		60–64	1 (4)
		65–69	5 (19)
		70–74	5 (19)
		75–79	7 (26)
		80–84	2 (7)
		85–89	1 (4)
		> = 90	1 (4)
Fall history and fear of falling	Experienced fall in previous year	Yes	14 (52)
		No	13 (48)
	Number of falls in previous year	0	13 (48)
		1	6 (22)
		2	4 (15)
		3+	4 (15)
	Whether fall(s) required medical attention^a^ (% among fallers)	Yes	8 (57)
		No	6 (43)
	Fall resulted in fracture (% among fallers)	Yes	3 (21)
	How worried are you about falling while walking or balancing?	1 Never	4 (15)
		2 Hardly	5 (19)
		3 Sometimes	11 (41)
		4 Often	4 (15)
		5 All the time	3 (11)
Current physical activity level	Currently engaged in some exercise group/activity^b^	Yes	19 (70)
		No	8 (30)
History of falls risk screening	Whether spoken to a GP or other professionals about risk of falling in previous year	Yes	11 (41)
		No	16 (59)
	If yes, where was it? (% among Yes for previous question)	GP	5 (45)
		Social care	0 (0)
		Falls clinic	3 (27)
		A&E	0 (0)
		Hospital	2 (18)
		Other	1 (9)
Falls prevention service use in past year	Type of falls prevention service use^c^	Physiotherapy	12
		Occupational therapy	1
		HAM	4
		Medication change	0
		Vision surgery	5
		Vit D supplement	6
		Assistive device	7
		Footwear change	6
		Falls education	12

Acronym: *HAM* home assessment and modification

^a^ At least GP visit

^b^ Suggested options were Chairobics, Pilates, dancing, swimming and group walks with additional space for participants to state other exercise/physical activity types

^c^ The list of services was taken from Cochrane systematic review of falls prevention trials [9]. However, the questionnaire did not explicitly label these services as falls prevention interventions in order to invite responses from participants who may have received a multi-purpose service (e.g., physiotherapy or vitamin D supplementation) without awareness of its falls prevention property. Overall, 21 participants (78%) indicated use of one or more service

Regarding current access to falls prevention, 11 reported having spoken to a professional about falls risk. Nevertheless, 21 reported recent use of services with some falls prevention properties [9], suggesting that the main falls prevention pathway under current practice is self-referral by older persons. Of the 21 users, 13 reported accessing multiple interventions. The most widely accessed services were physiotherapy and falls education.

### Framework (I): facilitators and barriers and cross-component and contextual factors

Table 2 summarises the identified facilitators and barriers to implementation by pathway component. The themes are numbered to facilitate re-mapping to later frameworks. Table A in Supplementary Material shows the direct transcript quotes for each theme. Figure B in Supplementary Material graphically illustrates how themes were mapped from qualitative data to Framework (I) and subsequently re-mapped to Frameworks (II) and (III).

**Table 2:** Summary of identified facilitators and barriers to the falls prevention pathway components

Pathway component [Thematic category #]	Facilitator [Thematic category #]	Barrier [Thematic category #]
*Falls risk screening and assessment by professionals* [1]	(A) Professional competence	
	• General approachability of professionals [1]	• Lack of proactive professional approach [1–5] • Lack of professional attention to environmental risk factors [1–6]
	(B) System-level approaches and resources	
	• Proactive, data-based approach to falls risk screening [1, 2] • Specialist expertise and equipment [1–3]	• Time constraint in routine practice [1–7]
	(C) Motivation and awareness of older persons	
	• Older person’s motivation to maintain health [1–4]	• Older person’s lack of falls risk awareness [1–8]
*Raising awareness of falls risk* [2]	• Awareness from earlier life-course stage [2-1] • Awareness of falls risk by informal caregivers [2]	• Lack of awareness of the physical ageing process [2, 3]
*Initial uptake of falls prevention treatments* [3]	(A) Motivation and awareness of older persons	
	• Older person’s experience of falling [3-1] • Older person’s experience of the physical ageing process [3-2] • Older person’s motivation to maintain health [3]	• Older person’s lack of falls risk awareness [3–15] • Low motivation of older persons [3–16]
	(B) Facilitators and barriers in the community	
	• Community marketing [3, 4] • Peer recommendations [3–5] • Marketing health benefits of interventions [3–6]	• Lack of information in community [3–17] • Barriers related to socioeconomic class [3–18] • Linguistic barriers to information uptake [3–19]
	(C) Intervention characteristics	
	• Intervention is free/cheap [3–7] • Intervention is enjoyable [3–8] • Intervention is of suitable difficulty [3–9] • Intervention is safe [3–10] • Intervention is conveniently located [3–11]	• High intervention cost [3–20] • Inconvenient timing of intervention [3–21] • Lack of safe venues for intervention [3–22] • Transport access and cost issues [3–23]
	(D) Professional competence and funding	
	• Professional recommendations are more important than peer recommendations [3–12] • Professional awareness of community initiatives [3–13] • Person-centred professional referrals [3–14]	• Lack of professional awareness of community initiatives [3–24] • Commandeering attitude of professionals [3–25] • Reactive professional approach [3–26] • Mismatch between area-based demand and supply [3–27]
*Adherence and long-term participation in falls prevention treatments* [4]	(A) Motivation and health of older persons	
	• Older person’s motivation to maintain health [4-1]	• Older person’s illness and comorbidities [4–10]
	(B) Positive and negative experiences of intervention characteristics	
	• Experience of intervention reducing falls risk [4-2] • Experience of wider health benefits of interventions [4-3] • Intervention is enjoyable [4] • Intervention enables high social participation [4, 5] • Intervention is individually tailored [4–6]	• High intervention cost [4–11] • Intervention is of unsuitable difficulty [4–12] • Intervention is not individually tailored [4–13] • Inconvenient timing of intervention [4–14] • Transport access issues [4–15]
	(C) Professional availability and competence and funding	
	• Availability of staff [4–7] • Proactive professional approach to sustain adherence [4–8] • Good professional-participant relationship [4–9]	• Lack of professional and volunteer staff [4–16] • Insufficient public sector funding [4–17]

#### Falls risk screening and assessment by professionals

Factors influencing falls risk screening and assessment by professionals could be divided into three groups: (A) professional competence; (B) system-wide approaches and resources; and (C) motivation and awareness of older persons. Participants were aware of the importance of professional competence in conducting the falls risk screening, particularly incompetence as barriers. For example, one participant had noticed the narrow scope of professional risk assessment:

(FG1) “I’d think it was important if somebody went to a health professional, the health professional would check on a whole lot of background information apart from immediate health thing – you know, what is your living, housing situation.” (Theme [1–6])

Nevertheless, participants were also aware of the impact of system-level approaches and resources beyond individual professional competence and made suggestions on improvement. One such suggestion was to adopt a proactive, data-based approach to risk screening akin to mass vaccination:

(FG1) “And with regards to hooking people in, when flu jab time comes up, we all get a text or a message or we get told that we need a flu jab. So, follow that lead, really. I’m sure there’s a record showing age groups and then tell them ‘Look, this service is available. Come on in!’” (Theme [1, 2])

Moreover, a few comments suggested that older person’s motivation to maintain health would facilitate professional efforts to discuss falls risk and prevention:

(FG4) “If I was at risk, I would be happy to talk to [the professionals]. Because I would be happy to take any advice on anything that keeps me good as possible for as long as possible, if that makes sense.” (Theme [1–4])

#### Raising awareness of falls risk

Participants generally recognised that falls risk awareness is a matter of understanding the ageing process, not only from a certain senior age but from earlier adult life stages. For example, one participant expressed the difficulty of staying aware of falls risks at home during the gradual ageing process:

(FG1) “Well, it happens so gradually, doesn’t it … when it is part of ageing and degenerative thing, it’s not like they go over night from being perfect to being in a wheelchair. It’s such a gradual thing. And you get used to stuff. You get used to the fact that the rug was curled up at the end.” (Theme [2, 3])

The role of informal caregivers in maintaining awareness of falls risk, particularly in the living environment shared with older persons, was also highlighted.

#### Initial uptake of falls prevention treatments

Factors influencing the initial uptake of treatments could be divided into four main groups: (A) motivation and awareness of older persons; (B) facilitators and barriers in the community; (C) intervention characteristics; and (D) professional competence and funding.

For (A), experiences of falls and increasing physical constraints associated with ageing were important catalysts for treatment uptake. That said, one participant declined to enrol in falls prevention despite an experience of falling and professional referral; the fall experience was thought to be the result of a specific situation (postprandial syncope) rather than a symptom of general vulnerability:

(FG4) “The only time I had fallen over is if I’m standing up suddenly. I go dizzy and I had a blackout and fall over. The nurse at the medical centres offered for me to go on a course to avoid falling. But I thought it wasn’t really necessary because I only fall in that situation. So I didn’t go on the course. I just have to be careful when I stand up.” (Theme [3–15])

For (B), the level of information on the treatment in the community – spread via marketing and peer recommendations – was an important determinant of uptake, while participants perceived socioeconomic and linguistic barriers in how the information is received and acted upon:

(FG3) “I think it’s the actual area, and I do actually think it’s class related in terms of whether people would actually get up and go to something even if it’s advertised, unless there’s somebody actually suggesting having it up in GP surgeries.” (Theme [3–18])

Important intervention characteristics included cost, enjoyability, suitable difficulty, safety, location, timing, support facilities (e.g., lack of handrail at venue entrance), and transport issues (availability and cost). Individuals considered whether the specific combination of these characteristics suited their preference and ability to pay. For example, one participant perceived modest private cost as an acceptable trade-off to enjoyability, while another perceived transport costs as a key main barrier:

(FG3) “I do think people would find the three odd pounds if they found [the intervention] absorbed them and really interested them.” (Theme [3–8])

(FG1) “And also, money and transport, not a lot of us can afford to go, because it’s usually, what, a fiver to get you where you want to go and back and return. Not a lot of people can afford to. When you are on universal credit or job seeker’s allowance and benefit, I think when you’ve got a disability like I have long enough. I think it should be like the over 60s [person was under 60], they have a bus pass.” (Theme [3–23])

Participants acknowledged the influential role of professionals in determining their treatment uptake, more influential than their peers according to theme [3–12]. The key steps were professional awareness of falls prevention initiatives in the community, followed by proactive recommendations or referrals made in a respectful and person-centred manner:

(FG1) “One person when we had a meeting found out that so many doctors were handing out too many drugs instead of an alternative. There was an alternative. [My doctor at surgery] said, ‘I’d want you to go and do an aquarobics’ and that helped me, that helped me so much that I didn’t need the drugs.” (Theme [3–14])

#### Adherence and long-term participation in falls prevention treatments

Factors influencing long-term treatment adherence could be divided into three main groups: (A) motivation and health of older persons; (B) positive and negative experiences of intervention characteristics; and (C) professional availability and competence and funding.

Significant illness or comorbidity impeded older persons’ adherence to interventions (theme [4–10]); but preventing an adverse health/functional status also served as a motivation for adherence:

(FG3) “Wanting to maintain what you’ve got. Not wanting to lose your independence. And hang on [to] independence as long as possible because I live alone as well.” (Theme [4-1])

Positive intervention experiences or characteristics that sustained adherence included falls risk reduction, wider health benefits, enjoyability, high social participation, and tailoring to individual ability. Negative ones included high cost, unsuitable difficulty, lack of tailoring, inconvenient timing and transport problems. Active involvement of healthcare professionals was not a guarantee that the intervention experience would be positive:

(FG3) “[The GP] set up [a programme] for people to stop falls. And I was in a group of about 8 people. And it was like a small version of going to the gym. And I went to that once and then I postponed it because it’s too hard for my hands.” (Theme [4–12])

Discontinuities in staff availability and funding unsurprisingly impeded long-term adherence. Otherwise, good bonding between the professional leader and participants was an important facilitator:

(INT1) “She [the Dance to Health instructor] goes out of her way to have friendly relationship with everyone that goes. And I think it works. You always get a cuddle when you arrive. And she always shows interest in you, what you are doing and what difficulties you have, and so on.” (Theme [4–9])

#### Cross-component factors

Two common themes across components were older persons’ health motives (themes [1–8, 4-1, and, 3-16]) and professional competence ([1–1, 1–5, 1–6, 3–12 to 3–14, 3–24 to 3–26, 4–8 and 4–9). First, older persons’ health-related goals such as maintaining independence facilitated risk screening by professionals ([1–4]), risk awareness ([2-1]) and intervention uptake ([3]) and adherence ([4-1]). Secondly, participants perceived that it is professionals’ responsibility to identify all relevant falls risk factors and prescribe relevant treatments (e.g., [1-6 and 3-14]); incompetence resulted in iatrogenic harm despite patient’s awareness:

(FG2) “I’ve got loads of medication variation problems. For me, I don’t really expect GPs to improve things, but they never told me ‘Oh we could change this into that’. He [the GP] just expects me to just keep pre-ordering the medications. So I leave it that way.” (Theme [3–26])

There was a close overlap in factors determining treatment uptake and adherence and long-term participation, both components sharing the themes concerning motivation of older persons, intervention characteristics and professional competence. As for factor differences, experience of falling was mentioned as a facilitator for uptake ([3-1]) but not adherence. Socioeconomic and linguistic barriers were mentioned only for uptake ([3-18 and 3-19]), likely because they are sufficient to discourage both uptake and adherence for the marginalised subgroups. Funding constraints impeded both uptake and adherence, though in different ways: adherence was predictably curtailed by the funding cut at the end of the pilot period ([4–17]); while uptake was impeded by deliberate policy to concentrate funding in deprived areas despite higher demand in well-off areas:

(FG3) “Now, to be honest, this [well-off] area doesn’t usually have anything. You know, I mean, all the money and the grant has been put into only deprived areas.” (Theme [3–27])

#### Contextual factors influencing the falls prevention pathway

Table 3 summarises the contextual factors that influenced the pathway implementation. They could be divided into two groups: (i) intersectoral factors; and (ii) prioritising the vulnerable groups. Table B in Supplementary Material shows the direct transcript quotes.

**Table 3:** Summary of contextual factors influencing the falls prevention pathway

Intersectoral factors [Thematic category 5]	Prioritising the vulnerable groups [Thematic category 6]
• Health hazards in local public spaces [5-1] • Health-promoting local public spaces [5-2] • Home ownership and modification [5-3] • Communitarian approaches [5-4]	• Persons with complex comorbidities [6-1] • Persons experiencing cognitive decline [6-2] • Socially isolated persons [6-3]

##### Intersectoral factors

Intersectoral factors concerned matters typically addressed outside the healthcare system, including the safety and health-promoting features of local public spaces, the relationship between home ownership and ability to implement home modifications, and potential communitarian approaches that mobilise the community to meet common goals. Older participants mentioned how in the past the local community would handle the challenges that lie outside the local/central government’s responsibility; the decline in communal responsibility was perceived to explain the increase in local health hazards:

(FG1) “I don’t think neighbours are neighbours anymore, either. When we were younger, I remember when snow came here, all the men of each family would come and make a path. And they don’t do that now.” (Theme [5-4])

##### Prioritising the vulnerable groups

Another set of themes concerned the need to prioritise the most vulnerable individuals at risk of a serious fall or loss of independence. Three groups were identified: persons with complex comorbidities; persons experiencing cognitive decline; and socially isolated persons. The reported experience of the diabetic participant who was below age 65 (hence below the eligibility age for the proactive pathway) illustrated how vulnerable individuals concurrently face multiple risk factors for serious falls:

(FG1) “If I had a bad day with my high sugar levels. I’ve had my bad day with blurriness. And I come down a lot of stairs and I fell X times coming down from attic and obviously coming out of my building which is a high old building. And then you’ve got to come down some more which is always full of leaves.” (Theme [6-1])

Despite this, public support for home assessment and modification was denied due to her ability to walk 100 m without problem, and support from other care professionals was similarly lacking.

### Framework (II): potential commissioning strategies

Table 4 re-maps the identified themes according to the CICI-HNA framework (see also Figure B in Supplementary Material).

**Table 4:** Themes arranged by the CICI-HNA framework to inform commissioning decisions

*Context, priority setting and need/eligibility [Theme #]*^a^	*Supply [Theme #]*	*Demand [Theme #]*
*Implementation context* • • Socioeconomic divide [3–18] • Linguistic divide/barrier [3–19] • Health hazards and opportunities in local geography [5-1, 5-2] • Legal/regulatory barriers for tenants to modify their homes [5-3] • Culture of communal responsibility that addressed key falls risk factors is no longer strong [5-4]	Provider and organisation • Positive professional attributes: approachable [1, 2]; aware of community initiatives [3–24]; proactive and person-centred care [3–14]; good relationship with intervention participants [4–9] • Negative professional attributes: reactive approach [1–26]; partial attention to risk factors [1–6]; commandeering attitude [3–25] • Facility/equipment: specialist Falls Clinics [1–3]; safe and well-located venues [3–23] • Positive intervention characteristics: low cost [3–20]; well-staffed [4–16]; enjoyable [3–8]; high social participation [4, 5]; suitable and tailored difficulty [3–13]; safe [3–10]; good timing [3–21]	Health and fall-related motives • Motivation to maintain health facilitates risk screening and uptake [1–6] • Previous experience of fall motivates uptake [3-1] • Experience of the physical ageing process motivates uptake [3-2] • Experience of intervention reducing falls risk and improving wider health motivates adherence [4-2, 4-3] • Lack of falls risk and ageing awareness impedes risk screening and uptake [1–15]
*Priority setting challenges* • Prioritising access for socially deprived and ethnic minority subgroups [3–19] • Prioritising access for vulnerable groups: complex comorbidities; cognitively impaired; socially isolated [6-1, 6-2, 6-3] • Where possible, needs of marginalised groups should be met without denying services to non-marginalised groups [3–27]	Funding and policy • Health promotion in earlier life course stages [2-1] • Use of routine data to facilitate risk identification [1] • Alleviating time constraints in care routine practice [1–7] • Funding to remove private intervention costs [3–20], sustained over the long term [4–17] • Auxiliary implementation strategies: information to informal caregivers [2]; community marketing [3–6]; peer health champions [3–5]	Psychosocial motives • Psychosocial benefits of interventions motivating uptake and adherence: enjoyability [3–8]; social participation [4, 5] • Good professional-participant relationship facilitates adherence [4–9]
*Need/eligibility* • Consider needs of chronically ill, frail and with comorbidities (who may be aged < 65) [4–10] • Identify appropriate interventions for cognitively impaired [6-2] • Consider targeting those living in vulnerable circumstances such as socially isolation [6-3]	Intersectoral policy • Improve public spaces: safer and more health-promoting [5-1, 5-2] • Change incentives for landlords to modify homes [5-3] • Make transport cheaper and more accessible [3–23] • Support community organisations and initiatives [5-4]	External influences on demand • Older persons are receptive to auxiliary implementation strategies, including community marketing and peer recommendations [3–6] • Older persons are particularly receptive to professional recommendations [3–14]

Acronym: CICI: Context and Implementation of Complex Interventions (CICI) framework [20]; HNA: Health Needs Assessment framework [43].

^a^ See Tables 2 and 3 for themes by falls prevention pathway component and Tables A and B in Supplementary Material for transcript quotes

#### Context, priority setting and need/eligibility

The first column of Table 4 groups together the themes on context, priority setting and need/eligibility. Not all contextual domains in the CICI framework were identified; the five identified were socioeconomic, linguistic/ethnic, setting/geographical, legal/regulatory and cultural. The commissioner and stakeholders should discuss to what extent the contextual factors are modifiable via intersectoral policies (i.e., lie within the decision space). For example, the difficulty of making safety modifications to rented properties was mentioned several times:

(FG4) “And I couldn’t [modify my house] because I live in a rented property. It’s not mine. I’m not allowed to do anything.” (Theme [5-3])

This could potentially be addressed by new housing regulations that incentivise relevant action by landlords. The culture of communal responsibility could be enhanced to some extent by supporting community organisations and civic initiatives.

Several priority setting challenges emerged from the data. The commissioner should consider prioritising intervention access for several marginalised subgroups: socially deprived; ethnic minority; with complex comorbidities; cognitively impaired; and socially isolated. Ideally, the prioritisation should not come at the expense of reduced services for non-marginalised subgroups.

The commissioner may also decide to change the eligibility criteria for falls prevention according to local priorities. Currently, CG161 recommends community-based falls risk screening for those aged 65 and over, followed by referral to multifactorial intervention for those at high falls risk defined by falls history and abnormal gait/balance. The screening protocol can be expanded to include those with complex comorbidities who are aged less than 65; the risk factors examined for referral can similarly be expanded to cover frailty and non-health factors such as social isolation. A separate pathway may be designed for cognitively impaired persons who require tailored support from dedicated organisations:

(INT2) “But with these walks which are organised by the Alzheimer’s Society is that there are qualified people leading the walks.” (Theme [4–7])

#### Supply

Older participants identified a broad range of supply-side issues and solutions at provider/organisation, funding/policy and intersectoral levels as shown in the second column of Table 4. The commissioner should determine which solutions lie within the decision space: e.g., certain professional attributes such as commandeering attitude may not be modifiable in the short run. Significant investments – e.g., a new Falls Clinics, changes to GP reimbursement schedule for risk screening – would similarly take time and be constrained by the budget.

#### Demand

The last column of Table 4 arranges the demand-side themes by three types: health and fall-related motives of older persons; non-health and social motives; and external influences on demand. Importantly, the external influences are modifiable by using auxiliary implementation strategies (e.g., community marketing). Older persons are also receptive of professional recommendations; hence, this influence can be maximised by improving professional attributes such as awareness of community initiatives:

(FG3) “When I was having as many as things I’ve had, I had to see Professor [name] at Hallamshire [Teaching Hospital]. So actually, I sent him details of [Dance to Health] and he wrote me to send me a very brief letter back saying ‘Thank you for this. I think I can put this to my other patients who have got a similar thing.’” (Theme [3–13])

### Framework (III): challenges for public health economic modelling

Table 5 summarises the methodological and evaluative challenges for falls prevention economic model identified from the qualitative data (see also Figure B in Supplementary Material).

**Table 5:** Methodological and evaluative challenges for falls prevention economic modelling

*Methodological challenges [Theme #]*^a^	*Evaluative challenges [Theme #]*
Capturing non-health outcomes and societal intervention costs • Model should capture social benefits of falls prevention interventions [3–8]. • Model should capture private intervention and transport costs [3–23]. • Model should capture any time opportunity cost to participants and informal caregivers: e.g., due to inconvenient timing or location [3–21].	Perspective, type of analysis and time horizon • Under CUA, the generic health utility measure such as EQ-5D may not fully capture social benefits of interventions [3–8]; the model should consider broader wellbeing measure (e.g., ICECAP-O [54, 55]) • Societal perspective is likely necessary to capture societal intervention costs [3–23]. • Long time horizons required to capture dynamic trajectories and evaluate system changes incurring large sunk costs (e.g., [1–3]).
Considering dynamic complexity • Model should incorporate dynamic trajectories of ageing and falls risk influencing older person’s demand and appropriate professional response [1–5]. • Model should capture the dynamic trajectories of variables that delineate vulnerable subgroups (e.g., cognitive status, frailty) [6-1, 6-2, 6-3]. • Model should capture wider health benefits of interventions beyond falls prevention [4-3]. • Model should incorporate seasonal changes in falls risk due to environmental risk factors [5-1].	Types of intervention scenarios evaluated • Main intervention scenario should incorporate: local eligibility criteria tailored to changing falls risk profile; multiple non-mutually exclusive intervention pathways; external evidence on interventions which have similar characteristics as those preferred by local older persons.^b^ • Intervention costing should incorporate: cost of risk identification; cost of auxiliary implementation strategies; fixed/sunk costs for major system changes; cost of additional resources to achieve full set of positive intervention characteristics; cost of professional training to obtain positive attributes; and funding to sustain intervention over sufficiently long period.^c^ • Additional scenarios conducting value of implementation analyses to evaluate auxiliary implementation strategies [2–6]. • Additional scenarios evaluating intersectoral policies (e.g., environmental interventions [5-1, 5-2]) and earlier life-course preventive interventions [2-1].
Considering theories/models of human behaviour based on psychology and sociology • Model should incorporate the health/social motives of older persons that influence demand [1–4] • Model should incorporate sociological and contextual factors that influence falls prevention: cultural factors promoting/weakening communal responsibilities for health promotion and safety [5-1, 5-2, 5-4]; regulatory barriers [5-3].	
Considering social determinants of health • Model should incorporate socioeconomic and ethnic/linguistic variables and social isolation as social determinants of health [3–19].	Analysis of equity and other priority setting criteria • Model should examine equity-efficiency trade-offs in adopting strategies that reduce social inequities of health [3–27] or prioritise other vulnerable groups [4–10].

Acronym: CCA: cost-consequence analysis; CUA: cost-utility analysis: ICECAP-O: ICEpop CAPability measure for Older people; NICE CG161: National Institute for Health and Care Excellence Clinical Guideline 161 [2]

^a^ See Tables 2 and 3 for themes by falls prevention pathway component and Tables A and B in Supplementary Material for transcript quotes

^b^ Local decision-maker could set the eligibility criteria for falls prevention referral, e.g., to cover those aged less than 65 who have complex comorbidities [6-1]. The intervention strategy should accommodate the changing falls risk profile that necessitates different treatments over time [1–5]. Non-mutually exclusive prevention pathways include: (i) proactive – involving referrals of high-risk older persons by professionals after risk screening as recommended by NICE CG161 [2]; (ii) self-referred – where older persons enrol in falls prevention without professional referral; and (iii) reactive – where older persons are referred to falls prevention by professionals after medical attention for a fall. Key intervention characteristics beyond cost are: staffing level [4–16]; enjoyability [3–8]; social participation [4, 5]; suitable and tailored difficulty [3–13]; safety [3–10]; and good timing [3–21]. External evidence (e.g., efficacy from randomised controlled trial) should be sourced from interventions with desirable characteristics

^c^ Cost of risk identification includes the cost of conducting risk screening in GP routine practice [1–7]. Auxiliary implementation strategies include information provision to informal caregivers [2], community marketing [3–6] and promotion of peer recommendations [3–5]. Major system changes include improvements to data systems [1] and new Falls Clinics [1–3]. Additional resources may be required to achieve the full set of positive intervention characteristics: e.g., hiring venues that are safe [3–22] and easy to reach [3–23]. Investment in training may increase the level of positive professional attributes including approachability [1, 2]; awareness of community initiatives [3–24]; person-centred care [3–14]; and relationship-building with intervention participants [4–9]. Funding should be sustained until the intervention has had enough time to generate substantial results [4–17]

#### Methodological challenges

The data identified several non-health outcomes (e.g., social benefits of group exercise) and societal intervention costs (e.g., private intervention and transport costs, costs of venues donated by local church) which were important facilitators and barriers. No older person mentioned time opportunity cost imposed on him/herself or his/her caregiver from attending interventions; but such costs may be incurred if interventions are conducted in inconvenient times and venues and should thus be incorporated in the model.

The dynamic processes of ageing and falls risk progression, starting before the age of 65, were mentioned by some participants as motivating factors for intervention uptake/adherence; yet others perceived the emerging illnesses as major barriers:

(FG4) “Well, I used to go swimming a lot every week. But then, since a long period of illness, I stopped going.” (Theme [4–10])

Either way, the model should seek to capture the dynamic trajectories of physical and cognitive capacities, functional status and health perception that determine the intervention demand and the composition of vulnerable subgroups. Moreover, the dynamic progression means that persons at different stages of the falls risk progression have different intervention needs; the model can quantify the added benefits of an intervention strategy that tailors treatments to progression stages relative to a strategy that does not. An example of the latter was perceived by older participants:

(INT2) “I think [the professionals] ought to check things like stairs and back steps. And not expect the elderly people to report it, because they are probably so used to these things when they’ve lived in the house all the time and are not necessarily aware of how less well coordinated they are from before.” (Theme [1–5])

Participants also highlighted wider health benefits of exercise beyond falls prevention, including improved mobility and mental health:

(FG2) “Lots of my family have noticed the difference in my posture, in my walk; things like, I used to struggle bending down, picking things up from the floor. It gets you down. It affects your mental health. So yeah, my family have noticed a huge difference.” (Theme [4-3])

Hence, the model should incorporate multiple simultaneous health effects of falls prevention exercise; if this proves too complex, then at least the fall’s impact on wider health and functional outcomes (e.g., on a multivariate frailty index [56]) should be incorporated to capture the full health benefits of falls prevention.

Finally, the model should incorporate key psychological and sociological factors identified from the qualitative data (e.g., health motives influencing demand) using relevant external quantitative data. Social determinants of health identified from the data included socioeconomic and ethnic/linguistic barriers to intervention access and social isolation as a marker of vulnerable subgroup.

#### Evaluative challenges

Given the range of non-health outcomes and societal intervention costs, the model evaluation should consider using a broader wellbeing measure and taking the societal perspective [54, 55]. The model time horizon should be sufficiently long to capture the dynamic trajectories of key variables and the full health impact of interventions; large sunk costs incurred by intervention may also be evaluated over a longer horizon.

Several intervention scenarios emerged from the data that should be evaluated under base case analysis and alternative scenario analyses. All three prevention pathways – proactive, self-referred and reactive – were mentioned in the data (see theme [1–5] for participant discussion of a reactive HAM receipt), and hence should be considered in the base case analysis. The main intervention scenario (compared to usual care under base case analysis) should incorporate interventions that have some or all of the positive characteristics (see Table 4) such as allowing individually tailored difficulty. Where external studies are used as data sources (e.g., RCT for efficacy), they should evaluate interventions with similar characteristics as the model scenario.

Intervention costing should incorporate not only the cost of intervention delivery but also the cost of auxiliary implementation strategies used to generate the given uptake and adherence; for the proactive pathway, the cost of professional risk screening and referral should be included. Major system-level changes (e.g., integrated data system for risk screening) would incur fixed/sunk costs which may be incorporated as annuitized overheads. Costs would be incurred if additional professional training (resources) is required to obtain positive professional attributes (intervention characteristics).

An alternative, heuristic method to directly incorporating psychological and sociological variables in the model is to conduct value of implementation analyses as alternative intervention scenarios [57]. Additional monetary value of hypothetical improvements in intervention uptake/adherence can be estimated without knowing what psychological or sociological factors contributed to the improvements. The additional value is the maximum amount that can be invested in auxiliary implementation strategies that produce the given improvements.

The lower intervention access for the socioeconomically deprived and ethnic minority subgroups would mean that the intervention is less cost-effective. A strategy that prioritises access for these groups to reduce social inequities of health (e.g., concentrating funding in deprived areas [theme 3–27]) would introduce an equity-efficiency trade-off. The model should parameterise the causal mechanisms to quantify the trade-off; the strategy would be accepted if stakeholders find the trade-off to be reasonable [36]. A similar process of equity-efficiency evaluation can be applied to other vulnerable subgroups identified, i.e., those with complex comorbidities and cognitive impairment.

## Discussion

This study explored older people’s views on facilitators and barriers for implementing the community-based falls prevention pathway recommended by NICE as well as broader themes on raising falls risk awareness, intersectoral initiatives and prioritisation of vulnerable groups. Participants included service users and non-users and those at high and low risks of falling. The study also explored how the identified themes can be mapped on to frameworks that can inform commissioning decisions via a de novo falls prevention economic model. It was thereby shown that the framework analysis approach [44] can flexibly accommodate diverse frameworks according to research aims.

The methods and results of this study contribute to the growing field of research exploring how qualitative evidence can be effectively used to inform health technology assessment (HTA) [40]. The recent NICE Decision Support Unit (DSU) report, for example, critiques the limited consideration of qualitative evidence in the current NICE HTA guideline (process and methods guideline 9; PMG9) and sees the use of established, purpose-specific frameworks – including the CICI framework – as a tool for accelerated and standardised incorporation of qualitative evidence in the HTA decision-making process [40]. This study showed that the CICI framework, despite its focus on supply-side conditions, can be applied to service users and eligible non-users. Previous qualitative studies have indeed shown that older people are sensitive to supply-side issues including cultural-linguistic context of intervention, professional attributes and intervention characteristics [45, 58–60], making their views highly relevant to commissioning decisions that must consider how the supply-side conditions are perceived and accepted by service users. This study facilitated attention on users’ perception and demand by supplementing the CICI framework with the HNA framework that conceptualises intervention access as an outcome of interactions between demand, supply and normative need. Such flexible adaptation of the CICI framework is encouraged by the framework developers [20]. Moreover, both the CICI framework developers and the DSU report focus on the application of CICI to qualitative and mixed-methods systematic reviews and not to primary qualitative research [20, 40]. By applying the framework to primary research, this study demonstrates the wider potential reach of the framework.

This study also showed that the primary qualitative research on service users can identify the key methodological and evaluative challenges to public health economic evaluation and thus function as a vital step within the conceptual modelling process [41]. Having identified the key causal mechanisms, the qualitative data can also identify the necessary group of stakeholders to modify them, and those not already involved in the project can subsequently be recruited. These are *ex-ante*, or prospective, applications of the qualitative evidence to inform the de novo model development. Yet *ex-post* application may be equally valuable: in England and Wales, clinical commissioning groups (CCGs) and local authorities are required to implement an intervention approved by NICE HTA within 3 months of the approval unless major local barriers to implementation can be identified (recommendation 1.5.1) [61]. The local qualitative evidence can identify such barriers and/or anticipate any major differences in the local cost-effectiveness and population-level outcomes relative to those predicted by the HTA. Moreover, the decision model underlying the HTA approval can be critiqued based on the methodological and evaluative challenges identified by the local qualitative evidence. If the model performs poorly in addressing the challenges, then a de novo model can be commissioned; the qualitative data would then be applied *ex-ante*. As mentioned, the *ex-ante* approach is more relevant for community-based falls prevention since no HTA has been conducted, and existing models [11, 39] do not adequately address the methodological challenges. The 2019 surveillance for the update to NICE CG161 (not yet published at the time of writing, August 2021) also mentions no plan for economic evaluation nor indeed for primary/secondary qualitative research with older persons [62].

The holistic approach to exploring the falls prevention facilitators/barriers identified two cross-component factors: health motives of older persons; and professional competence. The role of health motives in influencing older persons’ health behaviour has been debated in the literature. One study in Scotland found that older people are unlikely to participate in exercise for health reasons but rather for the social rewards; while another found that health motives (e.g., maintaining functional independence) help translate intentions into actual change in health behaviour [42]. This study found that health motives operate alongside the social rewards of interventions which corroborates the findings of a previous qualitative systematic review of older persons’ views [58]. CG161 similarly recognises both factors and recommends that care professionals provide information on the physical benefits of modifying falls risk to older persons and caregivers (recommendation 1.1.10.2), while also promoting the social values of interventions (1.1.9.2) [2]. The absolute and relative strengths of health and non-health motives impact the final combination of intervention characteristics and auxiliary implementation strategies: for example, strengthening the health motives would require well-framed health messaging [52], while addressing the social motives is a matter of better intervention design. Commissioners should nevertheless note the wide diversity of motives/preferences in the older population: one survey of 134 older persons, for example, found that 46% preferred to exercise alone versus 44% in a group [63]. Importantly, the group environment may be less preferred by marginalised social groups (theme [3–18]); alternative intervention types, such as home-based digital falls prevention exercise taken up at home [64], may be considered.

The importance of the second cross-component factor, professional competence, is affirmed by CG161 which recommends that all healthcare professionals regularly dealing with older persons “develop and maintain basic professional competence in falls assessment and prevention” (1.1.10.1) [2]. Yet older participants perceived external constraints placed even on competent professionals, including time constraints. This corroborates the findings from a previous survey of English GPs which specified insufficient consultation time and lack of allied health professionals in the community as the most prominent barriers to implementing CG161 [25]. Therefore, commissioning should comprehensively account for care system bottlenecks and carefully cost the solutions for their removal. One economic model, for example, incorporated the cost of a citywide falls risk screening that was assumed to operate like a cancer screening programme [65]. Costs that are fixed/sunk would interact with uptake rate to produce worse cost-effectiveness if uptake is inadequate [66] and economies of scale if uptake is increased [65]. Hence, models should accurately portray the cost structure (fixed vs. variable) to characterise the impact of implementation quality on cost-effectiveness. Aggregate population-level health and/or economic impact is another outcome largely determined by implementation; the NICE PMG9, for example, stresses the need to account for such impact in HTA decisions (see recommendations 5.12.3 to 5.12.7). Yet cost-per-unit ratios (e.g., incremental cost-effectiveness ratio) are often interpreted in isolation when using economic evidence for decision-making [67–69]. The final model informed by the qualitative evidence should present both ratio and aggregate outcomes so that the full impact of implementation quality could be quantified [70].

Less emphasised in CG161 but visible in the qualitative data (e.g., theme [4–16]) is the role of nonclinical professionals and volunteers who can substantially influence both supply and demand given their proximity to older persons in the community [71]: a pilot falls prevention scheme in Sheffield, for example, found that falls risk screening conducted at local community groups and lunch clubs significantly increased uptake [72]. It is hence critical to value the nonclinical and volunteer contributions; and value of implementation analysis offers a heuristic method to that end [57]. For example, one falls prevention model set in a Massachusetts community of population size 44,000 estimated that increasing falls prevention uptake from 50 to 75% would yield an additional $2.79 million which is the maximum amount that can be invested in community organisations to generate such uptake increase [73]. Such monetary value can be combined with qualitative data on demand-side influences to devise a cost-effective implementation strategy.

The methods used in this study are applicable to other geriatric health areas. One care strategy attracting policy attention is integrated care, designed to create “connectivity, alignment and collaboration within and between the cure and care sectors at the funding, administrative and/or provider levels” [74]. Since 2014 in England, the Better Care Fund obliges CCGs and local authorities to create a shared budget for health and social care and other public services, and also invests its own capital (£6.4 billion in 2019–20) to facilitate integration [75]. Such a strategy brings problems of implementation as diverse service components and teams are combined [76]; the empirical results for integrated care schemes are accordingly mixed [77, 78]. The holistic, cross-component qualitative investigation of the facilitators and barriers is likely critical for the schemes’ implementation. The contextual factors are similarly critical as the age-related physical decline increases the influence of the wider environment in determining intervention need and demand [79–82]. The key methodological and evaluative challenges must likewise be addressed by any economic model of geriatric public health interventions: for example, the social disparity in health status is a prominent feature of geriatric population and raises equity issues [83, 84].

### Strengths and limitations

The simultaneous coverage of three frameworks – cross-component factors, intervention-related causal mechanisms and public health modelling challenges – is a key strength of this study. As mentioned, qualitative research and economic evaluation are typically siloed with no interdisciplinary learning [39, 47, 48]. By contrast, this study explores how qualitative data can directly inform model-based economic evaluation.

The study nevertheless has limitations. The purposive sampling could have accounted for social categories such as area-level deprivation, particularly given the importance of social determinants of falls prevention access. The sampling was concentrated around older persons living near the Sheffield city centre, meaning that persons living in rural suburbs were under-represented. Falls prevention service users were recruited mainly from Dance to Health group exercise programme, meaning that other service types were under-represented. Only six participants (22%) reported no current/previous use of services with falls prevention properties, meaning that views of service non-users were under-represented. Moreover, the sampling did not distinguish between service non-users and those who had rejected falls prevention who would have had significantly different views. Informal caregivers’ views could have been elicited given their central role in facilitating falls prevention [85]. A final caveat is that conceptual modelling is incomplete without eliciting the views of commissioners and falls prevention professionals [41]. Accordingly, this study is part of a broader research project that engaged commissioners and professionals in the conceptual modelling.

## Conclusion

Better understanding of older persons’ health motives and higher professional competence can improve the implementation of the NICE-recommended falls prevention pathway. Older persons are sensitive to implementation causal mechanisms, meaning that their views can inform contextual and supply-side changes to promote falls prevention and wider health promotion. They are also important stakeholders who can inform the development of a complex public health economic model. The conceptual model informed by qualitative data can direct the gathering of quantitative evidence and ensure the structural validity of the final model used for local decision-making. Future commissioning projects should similarly employ qualitative research with service users as the first step towards operationalising a quantitative economic model of the decision problem.

## Supplementary Information

**Additional file 1.**

## Data Availability

Anonymised transcripts of the recorded focus groups and interviews used for data analysis are available from the corresponding author on reasonable request.

## Abbreviations

CCG: Clinical commissioning groups
CG: Clinical guideline
CICI: Context and Implementation of Complex Interventions
FG: Focus group
HAM: Home assessment and modification
HNA: Health Needs Assessment
HTA: Health Technology Assessment
INT: Interview
NICE: National Institute for Health and Care Excellence
PMG: Process and methods guideline
RCT: Randomised controlled trial
