# Supplementary material for: Qualitative research to inform economic modelling: a case study in older people’s views on implementing the NICE falls prevention guideline
Source: BMC Health Serv Res. 2021 Sep 28;21:1020. doi: 10.1186/s12913-021-07056-1 (PMC8479997; doi:10.1186/s12913-021-07056-1)
Supplement: Supplementary file 1 — Additional file 1. [file 12913_2021_7056_MOESM1_ESM.docx]

# Framework (II): Potential commissioning strategies


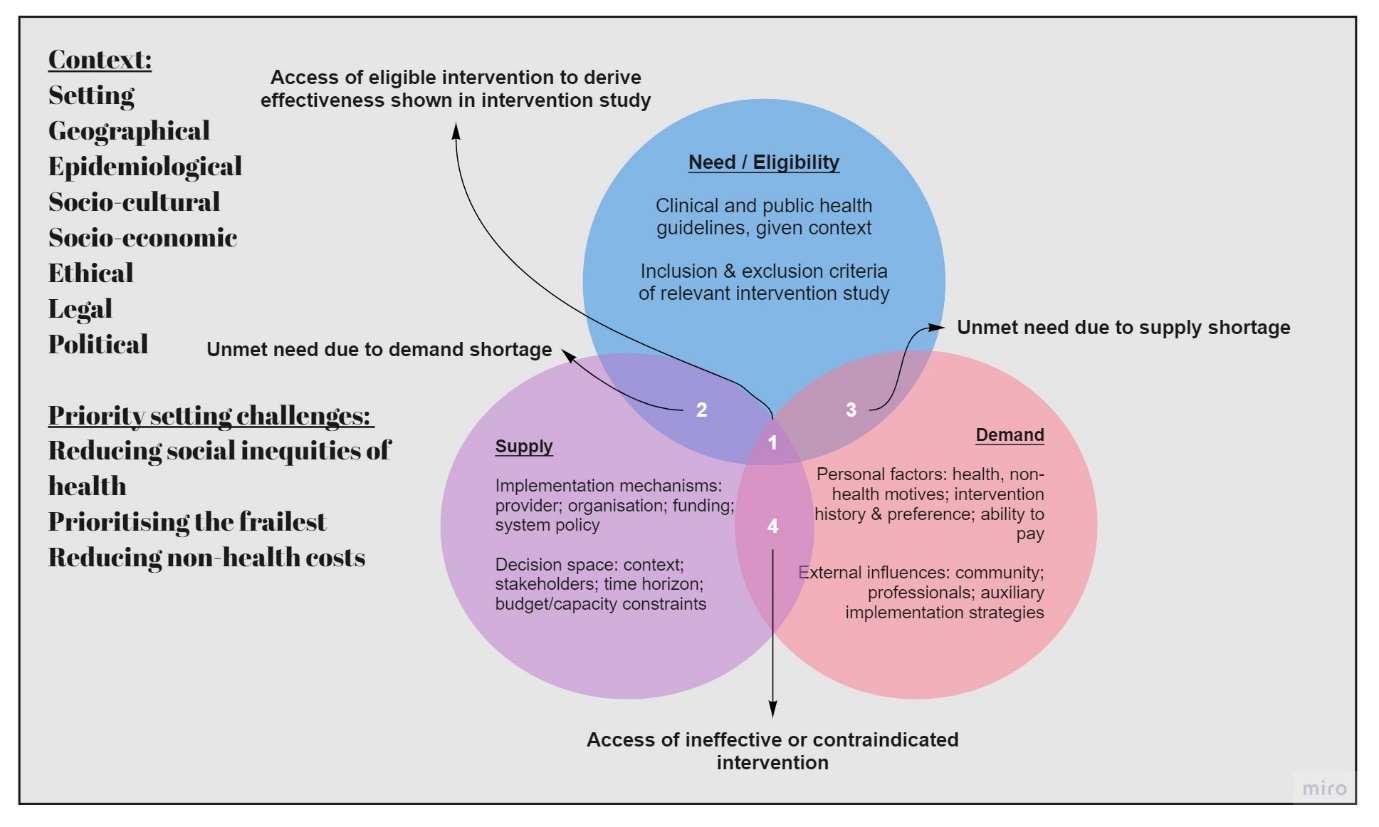


**Figure A** Combined CICI-HNA framework for organising qualitative data. CICI: Context and Implementation of Complex Interventions. HNA: Health Needs Assessment.

The CICI framework highlights eight domains of context, ranging from the immediate intervention setting to political influences (1). Context influences the implementation mechanisms of provider, organisation, funding and health system-wide policy, shown in the Supply circle of the HNA Venn diagram. Providers and organisations are micro- and meso-level entities delivering the commissioned interventions. Funding supports these entities as well as wider, auxiliary implementation strategies (e.g., community marketing to influence demand). Policies concern macro-, system-level changes to facilitate implementation (e.g., changes to GP reimbursement structure to facilitate regular falls risk screening).

The implementation context also presents priority setting challenges to the decision-maker. The three main priority setting criteria highlighted by an international panel of experts and stakeholders are reducing social inequities of health, prioritising the frailest and reducing the non-health costs (2).

The key consideration for formulating commissioning strategies is the decision space that defines which contextual factors and mechanisms are modifiable and to what extent. The decision space is determined by the combination of context and priority setting challenges, range of stakeholders involved, decision time horizon, and any budget and capacity constraints. For example, improving professional competence requires the cooperation of professional training institutions and may not be feasible in the short run; conversely, changing housing regulations may be feasible if the local Council and housing associations are actively involved in decision-making. The decision space may be largely pre-established prior to the qualitative study; alternatively, the qualitative findings may motivate changes to the decision space.

Intervention need/eligibility in the HNA Venn diagram is chiefly determined by normative clinical and public health guidelines and intervention studies that have used rigorous research designs to demonstrate the ability to benefit from the interventions (3). Yet, eligibility criteria may fall within the decision space if there is flexibility in how the criteria are applied in the local context. The CG161, for example, does not prescribe any specific care pathway for cognitively impaired persons (4); hence, the local commissioners and professionals may design a locally specific pathway. Framework (II) similarly seeks to identify major determinants of demand including personal factors underlying uptake/adherence decisions (e.g., health-related motives for healthy behaviour (5)) and external influences on demand (e.g., community marketing, self-efficacy promotions (6, 7)). The implications on commissioning are inferred from the types of demand-side factors and whether these fall within the decision space.

# Transcript quotes – pathway facilitators and barriers

| **Table A.** Transcript quotes for themes regarding facilitators and barriers to the falls prevention pathway components | | |
| --- | --- | --- |
| **Pathway component [Theme #]** | **Facilitator [Theme #]** | **Barrier [Theme #]** |
| Falls risk screening and assessment by professionals [1] | **General approachability of professionals [1-1]**  (FG4) “Well, I haven’t had an experience [of talking about falls], but I think my GP is very open for me to go and talk to them about it. I don’t think it would be a problem.” | **Lack of proactive professional approach [1-5]**  (INT2) “I think [the professionals] ought to check things like stairs and back steps. And not expect the elderly people to report it, because they are probably so used to these things when they’ve lived in the house all the time and are not necessarily aware of how less well coordinated they are from before.” |
|  | **Proactive, data-based approach to risk screening [1-2]**  (FG1) “And with regards to hooking people in, when flu jab time comes up, we all get a text or a message or we get told that we need a flu jab. So, follow that lead. I’m sure there’s a record showing age groups and then tell them ‘Look, this service is available. Come on in!’” | **Lack of professional attention to environmental risk factors [1-6]**  (FG1) “I’d think it was important if somebody went to a health professional, the health professional would check on a whole lot of background information apart from immediate health thing – you know, what is your living, housing situation.” |
|  | **Specialist expertise and equipment [1-3]**  (FG4) “[The Falls Clinic] is very impressive because you have a very detailed session with the professionals, and they ask you about all sort of things to do with your history. And from that, I got adaptation to my house. And I got the stick that I walk with.” | **Time constraint in routine practice [1-7]**  (FG4) “But after that fall, I went to the doctor, and they checked blood pressure and it was lower than required. But they didn’t give any medication. After that, about two weeks ago, again I started feeling I’m losing my balance at times [...] and I [went] to the doctors; but they only had 10 mins and they said they can’t check all these things but, at the same time, there was no problem [with balance].” |
|  | **Older person’s motivation to maintain health [1-4]**  (FG4) “If I was at risk, I would be happy to talk to [the professionals]. Because I would be happy to take any advice on anything that keeps me good as possible for as long as possible, if that makes sense.” | **Older person’s lack of falls risk awareness [1-8]**  (FG2) “Well, most of the people seem to…well, they haven’t got a problem of falling. They don’t consider it. So, they don’t sort of think about it.” |
| Raising awareness of falls risk [2] | **Awareness from earlier life-course stage [2-1]**  (FG1) “It’s not an over-65 issue. I’m under 65. I have issues. There’s quite a few of us here, I’m sure, that would benefit from [falls risk awareness].” | **Lack of awareness of the ageing process [2-3]**  (FG1) “Well, it happens so gradually, doesn’t it… when it is part of ageing and degenerative thing, it’s not like they go over night from being perfect to being in a wheelchair. It’s such a gradual thing. And you get used to stuff. You get used to the fact that the rug was curled up at the end.” |
|  | **Awareness of falls risk by informal caregivers [2-2]**  (INT1) “But my carer, who is a close friend, found [a non-protruding bed] for me on eBay. She could hardly get around the bedroom for cleaning. So she was quite aware that I needed to do something about sorting out the bedroom furniture.” |  |
| Initial uptake of falls prevention treatments [3] | **Older person’s experience of falling [3-1]**  (FG3) “I started coming [to falls prevention intervention] because of a fall. I had a dramatically awful fall and it’s just about a year ago. I was in hospital for 12 days and I had three carers a day for ages.” | **Older person’s lack of falls risk awareness [3-15]**  (FG4) “The only time I had fallen over is if I’m standing up suddenly. I go dizzy and I had a blackout and fall over. The nurse at the medical centres offered for me to go on a course to avoid falling. But I thought it wasn’t really necessary because I only fall in *that* situation. So I didn’t go on the course. I just have to be careful when I stand up.” |
|  | **Older person’s experience of the physical ageing process [3-2]**  (FG2) “Before taking up [falls prevention exercise], I was virtually stiff with arthritis and I didn’t get much exercise at all. And I was getting less and less…more relying on my husband to do everything.” | **Low motivation of older persons [3-16]**  (INT1) “[At the residence meeting] I talked about Dance to Health and what it is and said if anybody wants to come with me then just get in touch. But it hasn’t produced anybody. There just seems to be so much apathy now.” |
|  | **Older person’s motivation to maintain health [3-3]**  (FG4) “I will look for something because I know how important it is that I keep myself as healthy as I can possibly do.” | **Lack of information in community [3-17]**  (FG3) “I think that’s the biggest drawback. It’s getting people here. Because I can’t remember how I first heard [about the intervention].” |
|  | **Community marketing [3-4]**  (INT1) “Well, I always kept an eye out for the noticeboard in the sheltered scheme – and there was what you might call a flyer about the meeting for dancing in the city centre. I think it was at the library. So I went to that out of interest, and it turned out to be a flyer for the [falls prevention exercise] group I ended up with.”  (FG3) “I’ve started down this route through U3A, the University of the Third Age, which I am a member of.”  (FG3) “[Researcher: How can we increase uptake?] A poster in a library. Some sort of that will help. Or in the post offices. Somewhere people go.” | **Barriers related to socioeconomic class [3-18]**  (FG3) “I think it’s the actual area, and I do actually think it’s class related in terms of whether people would actually get up and go to something even if it’s advertised, unless there’s somebody actually suggesting having it up in GP surgeries.”  (INT1) “Group exercise in my view is quite a middle-class activity, and there isn’t much of a middle-class attitude here. One-to-one exercise might be most likely to get [non-middle-class individuals].” |
|  | **Peer recommendations [3-5]**  (FG3) “About two years ago, I was diagnosed with various diseases which did impact my balance quite considerably. A friend, a mutual friend, here today, suggested to me that something that might help would be Dance to Health. So, I came on and tried that.” | **Linguistic barriers to information uptake [3-19]**  (INT1) “Part of the problem is, some of the people here, I can’t communicate with anyway because they can’t speak English. And they don’t come to [the sheltered scheme residents’] meeting – no point coming if you can’t speak English.” |
|  | **Marketing health benefits of interventions [3-6]**  (FG1) “You have to make people realise the benefit of [exercise]. Especially the quality of their health. So you have to sell the benefit of exercise to the people. And make them realise that by doing that exercise it doesn’t only bring you the short-term benefits but medium- and long-term benefits.” | **High intervention cost [3-20]**  (FG3) “Alright, for us [exercise attendees], five pounds [per week] might be nothing, but if all of your expenses are coming from pension, five pounds is a lot!”  (FG1) “The government says everyone is obese and more exercises is needed and everything. And then there’s things like paying for swimming lessons.” |
|  | **Intervention is free/cheap [3-7]**  (INT1) “I mean we pay five pounds per session at [falls prevention exercise]. If you turn it around and say you get paid five pounds then that might be more interesting!” | **Inconvenient timing of intervention [3-21]**  (FG4) “Well, I know that there is a Pilates class here [local community centre]. It’s just not on the very good day for me.” |
|  | **Intervention is enjoyable [3-8]**  (FG3) “I do think people would find the three odd pounds if they found [the intervention] absorbed them and really interested them.”  (FG2) “Basically, I’ve always done dancing since I was 4. I’ve always danced. I wanted something that was going be suited my needs, really. Dance to Health seems to offer that. It really has made a huge difference to me.” | **Lack of safe venues for intervention [3-22]**  (FG4) “[The Pilates teacher] had a studio with stairs going to it and no handrail. And I just can’t, because I had my accident on steps, I really feel nervous at getting up at any steps without something that I can hold onto.” |
|  | **Intervention is of suitable difficulty [3-9]**  (FG2) “What kept me going was the fact that when I retired, a few people retired with me and they started in a [gym] group and things like that. And there was no way that I could’ve joined in anything like that. So, I came [to the falls prevention exercise group] instead.” | **Transport access and cost issues [3-23]**  (FG3) “If it’s planned properly and they are in the right places and people can get to them… this is the main thing that’s preventing people – people who would like to come but who can’t get here because they haven’t got their own transport.”  (FG1) “And also, money and transport, not a lot of us can afford to go, because it’s usually, what, a fiver to get you where you want to go and back and return. Not a lot of people can afford to. When you are on universal credit or job seeker’s allowance and benefit, I think when you’ve got a disability like I have long enough. I think it should be like the over 60s [person was under 60], they have a bus pass.” |
|  | **Intervention is safe [3-10]**  (INT2) “[At dementia-friendly walking groups] you’ve got people there who are ready to deal with an emergency.” | **Lack of professional awareness of community initiatives [3-24]**  (FG3) “What I think probably one of the problems is… like the health professionals, they don’t know what’s going on sometimes, do they?” |
|  | **Intervention is conveniently located [3-11]**  (INT2) “[The Pilates course is] just at the library around the corner and it is definitely meant for elderly people.” | **Commandeering attitude of professionals [3-25]**  (FG1) “So [the home visit professionals] came and they said ‘Right. We want. That carpet. We don’t want your rugs down. We don’t want that. You want another arm rail.’ And it invaded [my mother’s] space.” |
|  | **Professional recommendations are more important than peer recommendations [3-12]**  (INT1) “[Researcher: Do you think professional recommendation to exercise would be more effective than peer recommendation?] Yeah I think that might be more effective.” | **Reactive professional approach [3-26]**  (FG2) “I’ve got loads of medication variation problems. For me, I don’t really expect GPs to improve things, but they never told me ‘Oh we could change this into that’. He [the GP] just expects me to just keep pre-ordering the medications. So I leave it that way.” |
|  | **Professional awareness of community initiatives [3-13]**  (FG3) “When I was having as many as things I’ve had, I had to see Professor [name] at Hallamshire [Teaching Hospital]. So actually, I sent him details of [Dance to Health] and he wrote me to send me a very brief letter back saying ‘Thank you for this. I think I can put this to my other patients who have got a similar thing.’” | **Mismatch between area-based demand and supply [3-27]**  (FG3) “Now, to be honest, this [well-off] area doesn’t usually have anything. You know, I mean, all the money and the grant has been put into only deprived areas.” |
|  | **Person-centred professional referral [3-14]**  (FG1) “One person when we had a meeting found out that so many doctors were handing out too many drugs instead of an alternative. There was an alternative. [My doctor at surgery] said, ‘I’d want you to go and do an aquarobics’ and that helped me, that helped me so much that I didn’t need the drugs.” |  |
| Long-term adherence to falls prevention treatments [4] | **Older person’s motivation to maintain health [4-1]**  (FG3) “Wanting to maintain what you’ve got. Not wanting to lose your independence. And hang on [to] independence as long as possible because I live alone as well.” | **Older person’s illness and comorbidities [4-10]**  (FG4, Person 4) “Well, I used to go swimming a lot every week. But then, since a long period of illness, I stopped going.”  (FG4, Person 1) “I used to go swimming but I have a problem with IBS [Inflammatory Bowel Syndrome]. It’s one of the things that seems really hard to talk about, IBS, but it does affect one’s ability to exercise in all sorts of fields, particularly, swimming.” |
|  | **Experience of intervention reducing falls risk [4-2]**  (FG3) “Occasionally you’d feel you are going to fall over. You’d look drunk. But with doing [Dance to Health], it’s has now virtually gone and I haven’t had an attack over a year. I put it down the balancing techniques learnt in these classes. So, for me, with that particular condition, it’s been absolutely invaluable.” | **High intervention cost [4-11]**  (FG4) “Yes, I used to go to Pilates class, but I didn’t go for very long. But to my circumstances, I just couldn’t really afford to go. You know, five pounds an hour was a lot more than my budget allowed for.”  (FG1) “Everything comes down to costs. You can’t afford to go to gym or you can’t afford to keep [going to] fitness class. Everything comes down to whether you can afford to go.” |
|  | **Experience of wider health benefits of interventions [4-3]**  (FG2) “Lots of my family have noticed the difference in my posture, in my walk; things like, I used to struggle bending down, picking things up from the floor. It gets you down. It affects your mental health. So yeah, my family have noticed a huge difference.”  (FG1) “You’ve got satisfaction [from exercise] that you were feeling better without being pumped [with medications]. These exercises mean so much to me and it’s real.”  (FG3) “I gave up driving for a quite considerable time but now I have gone back driving because I feel I am a safe person on the road. And because of Dance to Health and these other factors, I now feel that my balance is… it’s not brilliant but it’s 500% better than it was.” | **Intervention is of unsuitable difficulty [4-12]**  (FG3) “[The GP] set up [a programme] for people to stop falls. And I was in a group of about 8 people. And it was like a small version of going to the gym. And I went to that once and then I postponed it because it’s too hard for my hands.” |
|  | **Intervention is enjoyable [4-4]**  (INT2) “Well people do tend to drop off if it’s boring stuff. I was given a sheet of exercises for home use but it’s boring. It’s much more pleasant in a group, you know.” | **Intervention is not individually tailored [4-13]**  (FG1) “If you go to like a leisure centre [Pilates] class or whatever, sometimes it might be 30 or 40 people in the room. And particularly if you’ve got a medical condition, they can’t give you that attention. You might be worried you are doing more harm than good, if they can’t direct correctly.” |
|  | **Intervention enables high social participation [4-5]**  (FG2) “A friendly group. It’s important. It’s not clinical. And it makes you want to come back and exercise.”  (INT1) “The other thing is the social side, the social contact. Everybody is obviously enjoying themselves and quite often says so. And so many good friendships being made. So there is a really active social side to it.” | **Inconvenient timing of intervention [4-14]**  (FG1) “And they altered the day [for Yoga classes]. So the day came and I couldn’t go anymore.” |
|  | **Intervention is individually tailored [4-6]**  (INT1) “If I’m having a bad morning, not moving all that well, I’m quite happy to tell [the instructor], and she’s quite happy to know that and take that into account when we do the exercises.” | **Transport access issues [4-15]**  (INT1) “Well, before I started taking the taxis regularly, I used to go by bus – and there were some difficulties. The bus didn’t always turn up and didn’t always stop.” |
|  | **Availability of staff [4-7]**  (INT2) “But with these walks which are organised by the Alzheimer’s Society is that there are qualified people leading the walks.” | **Lack of professional and volunteer staff [4-16]**  (FG1, Person 9) “And then this, um, Yoga teacher. She was an older lady. They sacked her because, they said, she was too old, and she was an absolute gem! They wanted a younger environment and person.”  (FG1, Person 1) “Volunteers have been cut down [for walking group]. A lot of volunteers have given up. We used to have two. But we only have one now. And if that one person is sick, then there won’t be a leader. Then we are just left walk ourselves. So the volunteers have [left]. They don’t get paid for it. They are just volunteering.” |
|  | **Proactive professional approach to sustain adherence [4-8]**  (FG3) “I think [the physiotherapist] was supposed to only come twice. But ended up with six times. And then she passed it onto a junior therapist. So, what they were doing was trying to check that I was doing it properly.” | **Insufficient public sector funding [4-17]**  (FG3) “They definitely got us [to enrol on falls prevention exercise] on the grant. So we didn’t pay when we started. There were a lot of pilot schemes all over Sheffield. And then the money ran out. We decided that we wanted to carry on and the church has been very supportive.”  (FG4) “I paid to go to physio privately because I will never be referred to go. I’ve got arthritis like everybody else. They are being referred to have physio on the NHS. They won’t continue with it. Whereas if you pay, you can go regularly.” |
|  | **Good professional-participant relationship [4-9]**  (INT1) “She [the Dance to Health instructor] goes out of her way to have friendly relationship with everyone that goes. And I think it works. You always get a cuddle when you arrive. And she always shows interest in you, what you are doing and what difficulties you have, and so on.” |  |
| **Acronym:** FG: focus group; INT: interview | | |

# Mapping, re-mapping and interpretation of themes across thematic frameworks


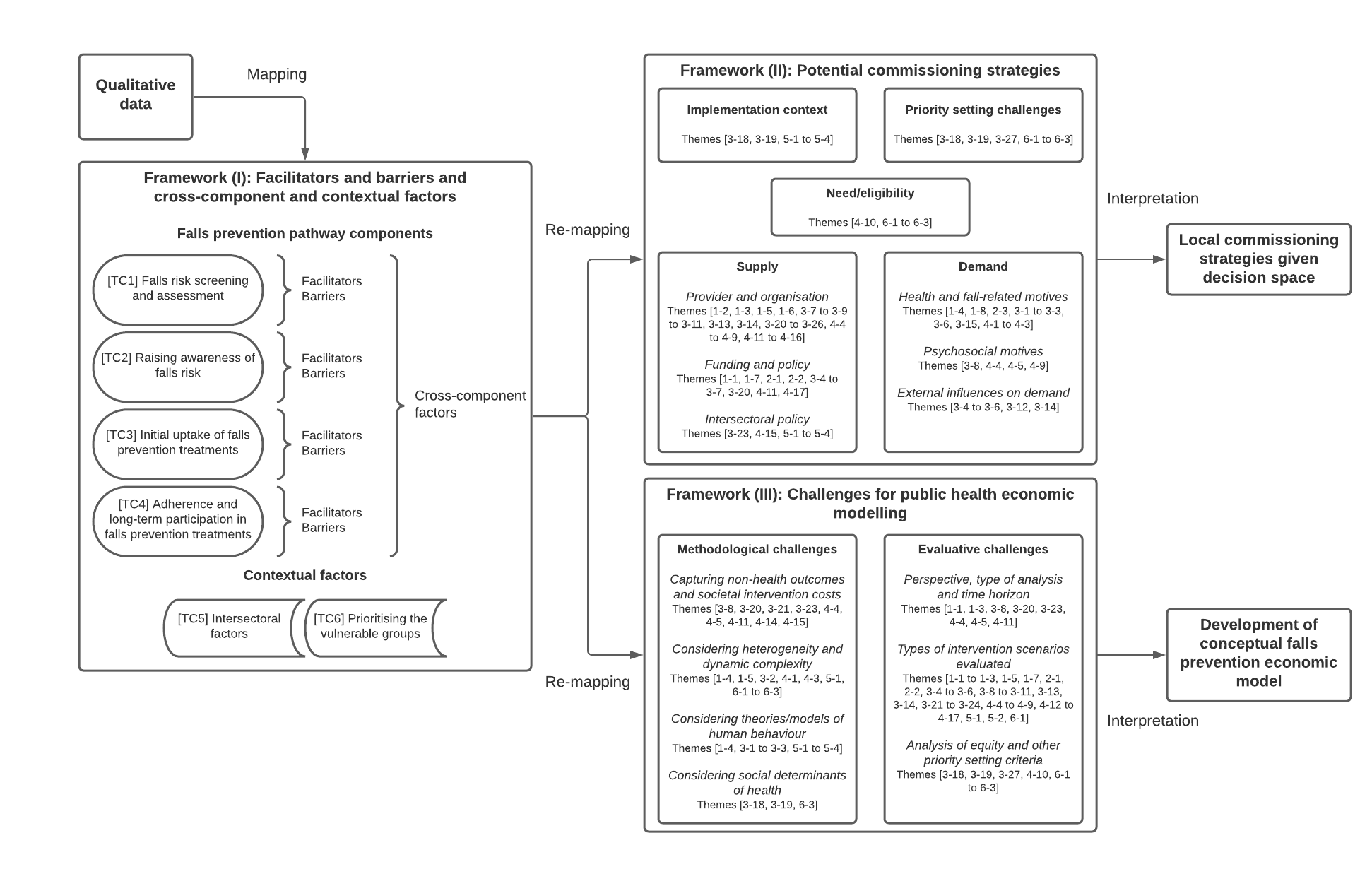


**Figure B** Themes mapped and re-mapped across three thematic frameworks and interpreted for commissioning and modelling. TC: thematic category.

# Transcript quotes – contextual factors

| **Table B.** Transcript quotes for contextual factors influencing the falls prevention pathway | |
| --- | --- |
| **Intersectoral issues [Theme #5]** | **Prioritising the vulnerable groups [Theme #6]** |
| **Safety concerns with local public spaces [5-1]**  (FG1) “And there’s lots of trees. And they all have shrubs cut. And they don’t sweep them up. And then they rot. And it’s right by the doctor’s surgery and I can’t tell you how many people have fallen there. People are frightened to go out in Autumn and Winter.”  (FG4) “We go up on the park and a lack of handrails on the steps. It’s just absolutely atrocious! There are certain steps which are quite steep, not handrails in sight.” | **Persons with complex comorbidities [6-1]**  (FG1) [Participant was aged below 65 and had diabetes-related complications that impaired her mobility, vision and mental health] “If I had a bad day with my high sugar levels. I’ve had my bad day with blurriness. And I come down a lot of stairs and I fell X times coming down from attic and obviously coming out of my building which is a high old building. And then you’ve got to come down some more which is always full of leaves.” [Despite this, public support was denied:] “I’ve just been through an [housing] assessment and it got turned down saying that I’m capable of doing everything for myself, saying ‘you can walk up to 100 meters without any problem’” [Other professionals were similarly disorganised:] “And I do mention [the falls risks] every time to my doctor and it’s the same thing every time: ‘Has anybody contacted you? Has a care worker contacted you?’” |
| **Health-promoting local public spaces [5-2]**  (FG1) “[In Hong Kong,] every building, at the bottom, they’ve got like an exercise machines and little gardens. And there’s old people doing exercises and they are getting together as well.” | **Persons experiencing cognitive decline [6-2]**  (INT2) [Participant was recently diagnosed with Alzheimer’s disease and experienced falls when she did not take her food supplements at the appropriate dose. But she repeatedly received lower dose and then faced difficulties in having the dose corrected:] “When it’s known that you have Alzheimer’s… People just shy off. You can very easily not be taken seriously if you make a fuss. You have to be very measured and careful in what you say.” |
| **Home ownership and modification [5-3]**  (FG4) “And I couldn’t [modify my house] because I live in a rented property. It’s not mine. I’m not allowed to do anything.”  (FG1) “Landlords haven’t really got time to be doing stuff like that [making sure stairways in house are safe.]” | **Socially isolated persons [6-3]**  (FG4) “I just think the main thing is I’ve got to try to make myself as healthy as I can, because you know, there’s only me to look after me. There isn’t anyone else. So, I’ve got to do best I can.” |
| **Communitarian approaches [5-4]**  (FG1, Person 5) “It would also be nice to raise… something like a funding for a charity so that we can all meet to raise motivation and participate.”  (FG1, Person 9) “I don’t think neighbours are neighbours anymore, either. When we were younger, I remember when snow came here, all the men of each family would come and make a path. And they don’t do that now.” |  |

# References

1. Pfadenhauer L, Rohwer A, Burns J, Booth A, Lysdahl KB, Hofmann B, et al. Guidance for the assessment of context and implementation in health technology assessments (HTA) and systematic reviews of complex interventions: the context and implementation of complex interventions (CICI) framework. Available from: <http://www.integrate-hta.eu/downloads/>: European Union; 2016.

2. Norheim OF, Baltussen R, Johri M, Chisholm D, Nord E, Brock D, et al. Guidance on priority setting in health care (GPS-Health): the inclusion of equity criteria not captured by cost-effectiveness analysis. Cost Effectiveness and Resource Allocation. 2014;12(1):18.

3. Stevens A, Gabbay J. Needs assessment needs assessment. Health trends. 1991;23(1):20-3.

4. National Institute for Health and Care Excellence. Falls in older people: assessing risk and prevention. National Institute for Health and Care Excellence. 2013;Clinical Guideline 161(nice.org.uk/guidance/cg161).

5. Schüz B, Wurm S, Warner LM, Wolff JK, Schwarzer R. Health motives and health behaviour self-regulation in older adults. Journal of behavioral medicine. 2014;37(3):491-500.

6. Ory M, Hoffman MK, Hawkins M, Sanner B, Mockenhaupt R. Challenging aging stereotypes: Strategies for creating a more active society. American journal of preventive medicine. 2003;25(3):164-71.

7. French DP, Olander EK, Chisholm A, Mc Sharry J. Which behaviour change techniques are most effective at increasing older adults’ self-efficacy and physical activity behaviour? A systematic review. Annals of Behavioral Medicine. 2014;48(2):225-34.
